# Walking Capacity in Parkinson’s Disease: Test–Retest Reliability of the 6-min Walk Test on a Non-Linear Circuit

**DOI:** 10.3390/healthcare14010018

**Published:** 2025-12-20

**Authors:** Asunción Mayoral-Moreno, Carlos Alexis Chimpén-López, Laura Rodríguez-Santos, María Isabel Ramos-Fuentes, José Carmelo Adsuar, Alejandro Caña-Pino

**Affiliations:** 1 Área de Psiquiatría, Facultad de Medicina y Ciencias de la Salud, Universidad de Extremadura, 06006 Badajoz, Spain; mmayoralm@unex.es (A.M.-M.); laura@unex.es (L.R.-S.); miramos@unex.es (M.I.R.-F.); 2 Área de Personalidad, Evaluación y Tratamiento Psicológico, Facultad de Enfermería y Terapia Ocupacional, Universidad de Extremadura, 10003 Cáceres, Spain; 3 Grupo de Investigación BioErgon, Facultad de Ciencias del Deporte, Universidad de Extremadura, 10003 Cáceres, Spain; jadssal@unex.es; 4 Red de Investigación Sobre Envejecimiento Saludable: Envejecimiento Activo, Ejercicio y Salud, Consejo Superior de Deportes (CSD), Ministerio de Cultura y Deporte de España, 28040 Madrid, Spain; 5 Grupo de Investigación Fisioterapia e Hipoterapia, Área de Fisioterapia, Facultad de Medicina y Ciencias de la Salud, Universidad de Extremadura, 06006 Badajoz, Spain; alejandrocp@unex.es

**Keywords:** physical function, 6 min walk, Parkinson’s disease

## Abstract

**Background/Objectives:** The 6 min walk test (6MWT) has been used to measure the level of walking capacity in people with Parkinson’s disease (PD). However, the use of a non-linear circuit has not yet been investigated deeply. Thus, the aim of this study was to evaluate the test–retest reliability of the 6 min walk test in a rectangular circuit in people with PD. **Methods:** Forty-two people with PD (men = 27, women = 15), mean age 66 ± 9.61 years, participated. All of them were in ON state. Each patient performed the 6MWT on 2 test days separated by 1 week, walking at a constant speed on a 20 m × 3.5 m circuit. Test–retest reliability was assessed using the intraclass correlation coefficient (ICC), minimal detectable change (MDC), and standard error of measurement (SEM), and Student’s *t*-test was applied to determine whether there were statistically significant differences between the test and the retest. **Results:** The ICC values for the total sample were excellent (>0.90) in both men and women. In addition, they had similar reliability, although slightly higher in women. The results showed that, for the total sample, a MDC < 17% can be considered as a true change for this procedure. The SEM percentage was 6.1%. **Conclusions:** The 6MWT, performed on a rectangular circuit, demonstrated excellent test–retest reliability in patients with Parkinson’s disease.

## 1. Introduction

Parkinson’s disease (PD) is a multifactorial neurodegenerative disease that involves the progressive deterioration of voluntary motor control [1]. It is the second most common age-related neurodegenerative disorder worldwide, affecting approximately 3% of adults over the age of 65 [2]. The risk of developing this disease is twice as high in men as in women, although women have a higher mortality rate and a more rapid disease progression [3]. Impaired voluntary motor control results in signs and symptoms such as bradykinesia, postural instability, rigidity and resting tremor, which commonly occur along with gait problems, arm, leg and trunk stiffness, and poor balance [4]. These problems have a negative impact on functional ability and activities of daily living [5].

The 6 min walk test (6MWT) assesses in an integrated manner the response of the cardiorespiratory, circulatory, muscular and blood systems that a person develops during exercise. The 6MWT measures the physical function necessary for a person to perform everyday activities [6]. The test assesses the maximum distance a person can cover on a flat surface for six minutes walking as fast as possible [7,8]. The test is widely used due to its simplicity and low cost [9], as it is performed at the subject’s own pace and does not require prior training or specific equipment [10]. It has been used in patients with various diseases including Parkinson’s disease [11,12,13,14,15,16].

Studies conducted with Parkinson’s patients have found that the average distance covered by patients in this test ranges from 391.6 ± 99.9 m to 465 ± 68.0 m [17,18]. Different studies confirm the variability that exists among patients according to motor severity and disease stage [19,20], freezing of gait, demographic and anthropometric characteristic, physical activity and fear of falling [17], quality of life [19] and balance and postural control [21].

It has been found that the 6MWT is commonly used to measure gait speed, as a measure of functional capacity, in people with gait limitations [22]. On the other hand, the 6MWT was strongly associated with established functional measures but was poorly associated with indirect measures of aerobic capacity. It seems that the test is more indicative of physical function and gait than endurance [23].

The 6MWT provides information on how motor symptoms affect the performance of daily activities, which is crucial for adjusting treatments and formulating rehabilitation strategies. In this regard, it offers objective details about gait quality and the possibility of being integrated into clinical evaluations to better define walking rehabilitation strategies in a quick and easy way [19,24].

Although the standard 6MWT protocol recommends a 30 m corridor [25,26], the scientific literature validates the use of alternative lengths and routes [27]. The space limitation, which often exists in the clinical environment, creates the necessity of validating this test on other courses. Furthermore, the use of a square or rectangular circuit could increase the frequency of motor challenges, which is very important to assess in patients with Parkinson’s. Turning is a complex task that intensifies difficulties with dynamic balance and is a known precipitant of freezing of gait and the risk of falls [28]. Although each turn in a rectangular circuit involves a smaller angle (approximately 90°) than the 180° turns in a standard linear course, the increased number of turns may cumulatively impose greater motor and balance demands, providing a relevant ecological challenge for people with Parkinson’s disease. The objective of this study was to evaluate the absolute and relative reliability of the 6MWT on a 20 m × 3.5 m rectangular circuit in PD patients, thus allowing for the determination of its potential as an effective tool to evaluate their walking capacity and exercise endurance in this population.

## 2. Materials and Methods

The study was approved by the Bioethics Committee of the University of Extremadura data 30 January 2023 (reference number: 120/2022). The authors assert that all procedures contributing to this work comply with the ethical standards of the relevant national and institutional committees on human experimentation and with the Helsinki Declaration of 1964, as revised in 2024. All participants signed the informed consent form.

This manuscript is part of a larger study in which other tests were evaluated. It was decided to publish the reliability of the tests separately, given that the objectives and hypotheses for each of these tests were different.

### 2.1. Sample Size

A single-group intraclass correlation design will be used to test whether the intraclass correlation is greater than 0.75 (H0: ρ ≤ 0.75 versus H1: ρ > 0.75). The comparison will be made using an intraclass correlation F-test, with a Type I error rate (α) of 0.15. To detect an intraclass correlation of 0.91 with 81% power, with 2 observations per subject, the number of subjects needed will be 14 (7 men and 7 women). The null and alternative hypotheses were established following the study by Koo et al. [29]. Although the calculation indicated that a minimum of 14 participants (7 men and 7 women) would provide sufficient statistical power (0.81%), a total of 42 participants were ultimately evaluated, increasing this statistical power to 99%.

### 2.2. Participants

42 patients with PD (men = 27, women = 15), with a mean age of 66.4 ± 9.61 years and an age range of 40–81 years, most commonly at stage II on the Hoehn and Yahr Scale, voluntarily participated in the test–retest reliability study. Participants were recruited by convenience sampling through local Parkinson’s disease associations and collaborating rehabilitation centres in the region of Extremadura (Spain). Information about the study was disseminated through association meetings and direct contact with physiotherapists involved in PD rehabilitation programmes.

Participants met the following:

Inclusion criteria: (1) Aged ≥ 18 years, (2) Diagnosed by a neurologist as stage I–III based on the Hoehn and Yahr Scale [30], and (3) No pathology contraindicating the physical exercise, for which the PAR-Q (Physical Activity Readiness Questionnaire) was administered [31].

Exclusion criteria: (1) Patients with PD in stages IV and V of the Hoehn & Yahr scale, (2) Patients with cognitive impairment and/or severe mental illness, (3) Having suffered a fall that makes it impossible to perform the 6MWT test, and (4) Ongoing psychiatric drug reactions, or other significant comorbidities.

### 2.3. Procedure

Participants performed the 6MWT in two sessions, one week apart. Both tests were conducted at approximately the same time of day (performed between 10:00 a.m. and 12:30 p.m.) to minimise variability caused by daily fluctuations in physical performance. The evaluation protocol of the 6MWT test was carried out with the participation of two evaluators who were actively involved in the first evaluation (Test) and the second evaluation (Retest). Each participant was evaluated by the same evaluator on both occasions. All participants were confirmed to be in the ON phase based on two criteria, patient self-assessment and evaluator observation, following a previously validated procedure [32]. Participants continued their usual therapies and medication regimens throughout the study period.

The one-week interval between the application of the 6MWT Test and the 6MWT Retest was selected to ensure the validity of the temporal reliability. This period is long enough to minimise the potential learning effect or memorisation of the performance by the participant. Simultaneously, the one-week window is short enough to ensure the stability of the clinical and physiological condition of the tested subjects, minimising the risk that observed variations in the 6MWT results are due to a real change in the participant’s functional capacity (e.g., disease progression or response to treatment) and not to measurement error inherent in the instrument [33].

Participants were instructed to perform the 6MWT using their usual assistive devices and it was ensured that the same device and method of support was strictly and consistently maintained at the test and retest. All tests were conducted outdoors on a flat, hard asphalt surface within a closed, quiet parking area free of vehicular traffic or pedestrian interference. The area was clearly demarcated using cones to define the 6 m straight and rectangular circuits. Ambient conditions (temperature ≈ 22 °C, no wind, adequate lighting) were consistent across sessions. No external auditory or visual stimuli were present, minimising potential environmental influence on gait performance. They walked at a constant speed for 6 min in a 20 m × 3.5 m circuit marked by four cones located at the ends; on reaching each cone the participant had to go around it and continue walking until they reached the next cone, and so on until the six minutes were up. At the end of the test, the total number of laps completed, plus the metres of the last lap, were added together (1 lap = 47 m). The test was carried out outdoors, on a flat surface, and participants were shown beforehand how to carry it out, by completing a full lap of the circuit from the starting point. During its development there were two evaluators to supervise the execution of the turns in the circuit and the test. Throughout the test, participant safety was prioritised as the two trained evaluators remained alongside the circuit to provide immediate support in case of imbalance or fatigue, and the test was stopped if any participant showed signs of instability, dizziness, or excessive exertion.

Before starting the 6MWT test, participants were given the following instructions: “The aim of the test is to walk as fast and as far as possible for 6 min. You must not run or jog; you must maintain a steady walking pace. Walk around the marked circuit. The examiner will count aloud for each complete lap. It is important to turn around the cone so that the route is as efficient as possible. If you need to stop or slow down, you may do so, but you are encouraged to resume your walking pace as soon as possible, maintaining maximum effort throughout the test. The examiner will give a countdown warning when there is one minute left until the end of the test. When the 6 min are up, the command ‘Stop!’ I will be given it. At that point, you must stop immediately and wait without moving until the exact distance from your stopping point is recorded”.

### 2.4. Clinical Evaluation

Sociodemographic. Participants completed a questionnaire focusing on age, gender, marital status, educational level, employment status, living arrangements, time of diagnosis of the disease, psychopharmacological medication and frequency of falls in the last 8 weeks and last year.

Self-perception of fitness. The International Fitness Scale (IFIS) was used [34]. This questionnaire assesses how participants perceive their general fitness, muscle strength, speed, cardiorespiratory fitness, agility and flexibility. The assessment instrument uses a five-point Likert-type scale, where the response options are graded from 1 (‘Very bad’) to 5 (‘Very good’). The total score is calculated as the direct sum of the scores obtained on the five items assessed. The possible range of scores is between a minimum of 5 points (obtained by selecting “1” for all questions) and a maximum of 25 points (obtained by selecting “5” for all items). A higher final score is interpreted as an indicator of a more positive and favourable self-perception of the participant’s overall physical fitness.

Quality of Life. This was assessed using the Parkinson’s Disease Questionnaire PDQ-8 [35]. It is a short form of 8 items present in the PDQ-39 that has been shown to be valid and reliable. It covers the following areas: Mobility, activities of daily living, emotional wellbeing, stigma, social support, cognition, communication and bodily discomfort. The score is obtained by adding up the responses (each item is rated from 0 to 4) and converting the result into a percentage, where higher values indicate a greater negative impact on quality of life.

Fear of Falling. Participants completed the Falls Efficacy Scale International (FES-I) [36]. This is a questionnaire that measures the level of concern about falling in sixty-six social and physical activities. The instrument consists of 16 items and is scored using a 4-point Likert scale, 1 ‘Not worried’, 4 ‘Very worried’. The total score is obtained by summing the responses. The possible score range is from 16 points (minimal or no concern) to 64 points (maximum concern). A higher score indicates a higher level of fear of falling.

### 2.5. Statistical Analysis

Pass 2025 Power Analysis & Sample Size (https://www.ncss.com/software/pass/, accessed on 27 October 2025) was used for statistical power and for sample calculations. Statistical analyses for Mac were conducted using Jamovi software Version 2.3.28.0, SPSS 23, and Excel 2024.

The Shapiro–Wilk test was conducted to assess the normality of all continuous variables. Variables that followed a normal distribution were described using mean ± standard deviation. Student’s *t*-test was used to determine whether there were statistically significant differences between the test and retest, as well as between sexes. The chi-square test was applied to examine sex differences in categorical variables. The significance level was set to *p* < 0.05. Reliability was examined using both relative and absolute reliability measures. Relative reliability was assessed through the intra-class correlation coefficient (ICC_3,1_) [37]. ICC data were calculated using the following parameters: (1) Model: 2-way random effects; (2) Type: single rater and, and (3) Definition: consistency [29]. The following classification was used for interpreting the ICC [38]: an ICC less than 0.5 corresponds to poor reliability, an ICC from 0.5 to 0.75 corresponds to moderate reliability, an ICC from 0.75 to 0.9 corresponds to good reliability, and an ICC greater than 0.9 corresponds to excellent reliability. Absolute reliability was determined using the standard error of measurement (SEM) and the Minimal Detectable Change (MDC). The SEM was calculated with the formula SEM = SD·$\sqrt{1-ICC}$; where SD is the mean SD of the two repetitions. The MDC formula was MDC = 1.96·SEM·$\sqrt{2}$. This score was subsequently converted to a percentage. For the interpretation of the MDC results, a change in the distance covered in the Six-Minute Walk Test (6MWT) of less than 20% from baseline will be considered a low variation. This approach is based on evidence suggesting that the Minimal Detectable Change (MDC) for the 6MWT in populations with parkinsonism is 82 m, which, for an average walking distance of approximately 400 m, is equivalent to a change of 20.5% [39,40].

To assess the level of agreement between test and retest regarding distance of the 6MWT, a Bland–Altman analysis was performed. In these plots, the *x*-axis represents the mean of the test, and the *y*-axis shows the difference between the two measurements (A–B; A = test; B = retest). Bias and 95% limits of agreement (LOA) were calculated. Bias values close to zero represent strong agreement and the smaller range between these two LOA are interpreted as better agreement [41]. This analysis also provides information on the presence or absence of systematic bias by evaluating whether the mean difference between measurements deviates significantly from zero.

Additionally, correlation analyses were performed to explore the relationships between the 6MWT distance and selected demographic and clinical variables (age, disease duration, quality of life, physical fitness, fear of falling, weight, and height). Depending on data distribution, Pearson’s correlation coefficient was used for parametric variables and Spearman’s rho for non-parametric variables. Statistical significance was set at *p* < 0.05.

## 3. Results

Table 1 and Table 2 include clinical and socio-demographic characteristics, for the total sample and men’s and women’s sub-groups. The mean age of participants was 66.4 ± 9.61, men 66.8 ± 9.57 and women 65.7 ± 9.97 (*p* = 0.72). Most participants were in Hoehn & Yahr stage II (54.8%), followed by stage I (31.0%) and stage III (14.3%), indicating that the sample primarily consisted of individuals with mild to moderate Parkinson’s disease.

No significant sex differences were found in any of the variables related to participants’ characteristics, except for height and fear of falling (Table 1). No statistically significant differences were found either in the sociodemographic variables between men and women, except in terms of employment status (Table 2).

Table 3 shows the descriptive data of the test and retest measures. Significant differences were observed for all participants regarding distance for 6MWT. In the men’s and women’s sub-groups, there were only significant differences for distance for 6MWT in men.

Table 4 shows relative reliability (ICC) and absolute reliability (SEM, SEM%, MDC, and MDC%). Total sample ICC values were excellent (>0.90) for all participants and men and women sub-groups. The ICC for the whole sample was 0.94, while the ICC for the men’s subgroup was 0.91 and the women’s subgroup 0.96. SEM% was 6.1 for all participants; MDC%, however, was 17. In the men’s sub-group, SEM% was around 6.1% and MDC% was around 17%. In the women’s sub-group SEM% was around 6.0 and MDC% was around 16.7%. The ICC was slightly better for the women’s sub-group. The results obtained show that the 6MWT is sensitive in the Parkinson’s population, with a detection in the lower measurement error (MDC < 17%).

**Figure 1:**
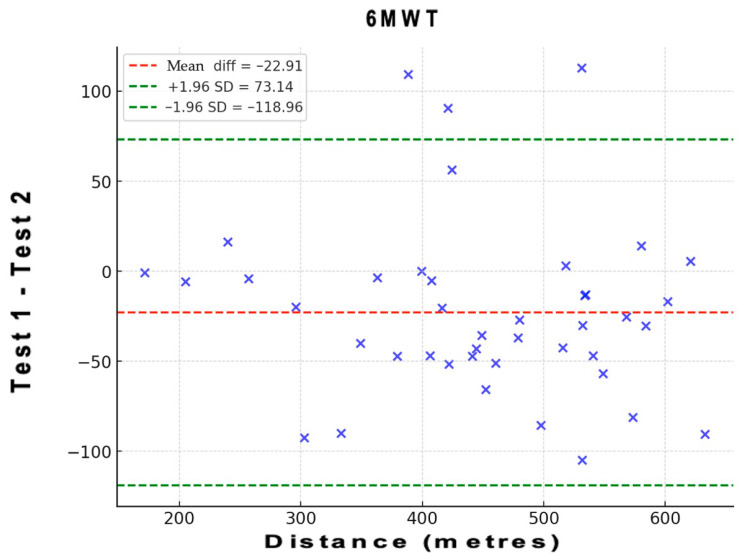
Bland–Altman plots of the 6MWT.

Figure 1 shows the Bland–Altman plots of the 6MWT. These plots indicate that the points outside the 95% LOA are less than 7.5%. The Bland–Altman analysis revealed a mean difference (bias) of 22.91 units between test and retest values, with 95% limits of agreement ranging from [−118.96] to [73.14]. The mean bias line was close to zero, and the data points were symmetrically distributed around it, indicating the absence of systematic bias. These results confirm good agreement between the two measurement occasions.

Correlation analyses were performed to explore associations between the 6MWT–T1 distance and selected demographic and clinical variables (Table 5). Height (r = 0.58, *p* < 0.001) and self-reported physical fitness (r = 0.34, *p* = 0.03) showed significant positive associations with the 6MWT distance, while age demonstrated a significant negative relationship (r = −0.36, *p* = 0.02). No significant correlations were found with disease duration, quality of life, fear of falling, or body weight.

## 4. Discussion

The present study confirms the excellent test–retest reliability of the 6MWT in patients with Parkinson’s disease, even when performed on a non-linear rectangular circuit of 20 × 3.5 m. This design requires greater number of turns than the standard protocol, which imposes a high motor and balance demand. In PD, the turning movement is a known precipitant of bradykinesia, difficulty in initiating movement, and freezing of gait, factors that typically increase intra-subject variability [28].

The 6MWT has proven to be a cornerstone in functional assessment across various pathologies [11,12,13,14,15,16]. Our findings confirm its applicability in PD patients. This study also examined the relationship between walking performance and selected demographic and clinical variables, including age, physical fitness, fear of falling, and quality of life, to provide a broader context for interpreting individual variability. Correlation analyses revealed that height and self-perceived physical fitness were positively associated with walking performance, while age showed a negative relationship. These findings are consistent with previous studies highlighting the influence of anthropometric and fitness-related factors on gait capacity in PD [16,22]. Interestingly, fear of falling and quality of life did not show significant associations, suggesting that objective performance measures may capture aspects of functional capacity not fully reflected in self-reported outcomes.

The mean distance covered in our study is consistent with the existing literature, approaching the values reported by Falvo and Earhart (391.6 ± 99.9 m) in mild to moderate PD [16] and those by Kobayashi et al. (340.8 ± 110.9 m) in moderate PD [42].

However, the high ICC suggests that, even under this increased demand, the walking performance was highly reproducible. This supports the use of shorter circuits not only for evaluating aerobic endurance but also as an ecological and functional challenge that more faithfully simulates the mobility demands in restricted home and community environments, which is fundamental for fall prediction.

The low MDC (≤17%) observed for the total sample indicates that a change in distance covered exceeding this percentage can be considered a true functional change, which is crucial for monitoring disease progression or the impact of therapeutic interventions in clinical practice. The comparable reliability between men and women further strengthens its universality within the PD population. An MDC of 17% can be considered low, indicating good measurement precision, as MDC values below 20% are typically interpreted as reflecting minimal measurement error and high sensitivity to change [40].

On the other hand, our results are also in line with this study by Steffen and Seney [40], who reported excellent test–retest reliability (ICC > 0.90) for the 6MWT in individuals with Parkinsonism (Hoehn & Yahr stages I–IV) with a MDC around 26%. Although their MDC value was larger than the 17% observed in our study, both investigations consistently demonstrate that the 6MWT is a highly reliable tool for assessing functional capacity in Parkinson’s disease. The lower MDC in our sample may be partially explained by methodological differences, including the use of a rectangular circuit, a shorter retest interval, and a relatively homogeneous sample restricted to Hoehn & Yahr stages I–III. The variability observed in the individual results can be attributed, in addition to the clinical variables specific to PD (age, gender, disease stage), to non-motor factors, such as the participants’ level of prior physical activity and their psychological state [17,18,19,20,21].

Despite the solidity of the findings, this study presents certain limitations. The inclusion of subjects exclusively in Hoehn and Yahr stages I–III requires caution when extrapolating the results to patients with more severe involvement. Furthermore, this investigation did not analyse the effect of medication on 6MWT performance. Therefore, future research should take these limitations into account, including analysing the results of the 6MWT in relation to the stage of the disease and the frequency of falls. It would be of interest to conduct longitudinal studies to determine the evolution of functional capacity and the impact of interventions. Likewise, it is recommended to incorporate complementary measures before or during the test, such as blood pressure, oxygen consumption, and heart rate. We believe this would provide a more complete picture of functional performance and the physiological response to exercise in patients with PD. In addition, the present study did not include concurrent validity testing.

Future research should examine correlations between the rectangular-circuit 6MWT and the standard linear 6MWT, as well as with other objective measures of physical function, since the present study only included concurrent validity tests between the 6MWT test and self-perceived physical fitness. It also would be interesting to compare results in 2MWT with the 10-Metre Timed Walking Test and the Timed Up and Go Test. Further studies are needed to examine its validity in relation to other established measures of functional capacity in Parkinson’s disease.

## 5. Conclusions

The rectangular-circuit 6MWT demonstrates high test–retest reliability, indicating that the protocol provides consistent results across repeated measurements.

## Figures and Tables

**Table 1:** Characteristics of the study participants.

		Total (*n* = 42) Mean ± SD	Men (*n* = 27) Mean ± SD	Women (*n* = 15) Mean ± SD	*p* *
Height (m)		1.62 ± 0.08	1.72 ± 6.46	1.55 ± 8.14	<0.001
Weight (kg)		78.6 ± 15.0	81.8 ± 11.7	72.7 ± 18.5	0.06
Time since diagnosis (years)		7.58 ± 5.18	7.61 ± 5.54	7.53 ± 4.64	0.96
Quality of life PDQ-8 (score 0–32)		8.48 ± 4.33	8.11 ± 4.14	9.13 ± 4.73	0.47
Physical Fitness Self-Assessment IFIS (score 0–25)		13.3 ± 3.16	13.6 ± 3.27	12.8 ± 2.98	0.42
Fear of falling FES-I (score 16–64)		30.6 ± 9.37	27.5 ± 8.73	36.1 ± 8.08	<0.001
		Total (*n* = 42)	Men (*n* = 27)	Women (*n* = 15)	p **
Stages of a disease (levels) (Hoehn & Yahr)	I	31%	26.2%	4.8%	0.08
	II	54.8%	33.3%	21.4%	
	III	14.3%	4.8%	9.5%	
Frecuency of Falls	“None”	61.9%	42.9%	19%	0.06
	“Occasionally”	21.4%	16.7%	4.8%	
	“Less than once a week”	14.3%	2.4%	11.9%	
	“Once a day”	2.4%	2.4%	0%	
	“More than once a day”	0%	0%	0%	
Psychopharmacological treatment	“Yes”	71.4%	45.2%	26.2%	0.75
	“No”	28.6%	19%	9.5%	

* Student’s *t*-test; *p* < 0.05: statistical significance; m: metres; kg: kilograms; ** Chi-square; *p* < 0.05: statistical significance; SD: Standard Deviation.

**Table 2:** Socio-demographic characteristics.

		Total (*n* = 42)	Men (*n* = 27)	Women (*n* = 15)	*p* *
Civil Status	“Married”	71.4%	50%	21.4%	0.59
	“Single”	11.9%	7.1%	4.8%	
	“Divorced”	11.9%	4.8%	7.1%	
	“Widowed”	4.8%	2.4%	2.4%	
Level of Education	“University”	14.3%	11.9%	2.4%	0.39
	“High School	19%	16.7%	2.4%	
	“School graduate”	45.2%	23.8%	21.4%	
	“No studies”	21.4%	11.9%	9.5%	
Employment Status	“Active”	19%	14.3%	4.8%	0.01
	“Retired”	69%	50%	19%	
	“Unemployed”	2.4%	0%	2.4%	
	“Homemaker”	9.5%	0%	9.5%	
Family Status	“Lives alone”	11.9%	4.8%	7.1%	0.10
	“Lives with partner”	64.3%	50%	14.3%	
	“Living as a family”	4.8%	2.4%	2.4%	
	“Others”	19%	71.1%	11.9%	

* Chi-square; *p* < 0.05: statistical significance.

**Table 3:** Data of the test-retest of 6MWT.

6MWT (m)			
	Day 1	Day 2	
Test Measurement	Mean ± SD	Mean ± SD	*p*
All participants	436.92 ± 113.06	459.83 ± 119.40	0.004
Men	470.72 ± 102.49	499.44 ± 99.45	0.007
Women	376.08 ± 108.43	388.55 ± 121.97	0.300

SD: Standard Deviation.

**Table 4:** Relative and absolute reliability of 6MWT.

Total (n = 42)	6MWT (m)				
Assessed Action	ICC (95% CI)	SEM (m)	SEM (%)	MDC (m)	MDC (%)
6MWT (m)	0.944 (0.897–0.970)	27.50	6.1	76.22	17
Men (n = 27)	0.913 (0.810–0.960)	29.78	6.1	82.55	17
Women (n = 15)	0.960 (0.885–0.987)	23.04	6.0	63.86	16.7

ICC: intraclass correlation coefficient; MDC: minimal detectable change; SEM: standard error of measurement.

**Table 5:** Correlations between 6MWT–T1 and demographic or clinical variables.

	r	*p*-Value
Age (years)	−0.36	0.02 *
Height (m)	0.58	<0.001 *
Weight (kg)	0.04	0.81
Time since diagnosis (years)	−0.05	0.76
Quality of life PDQ-8 (score 0–32)	0.10	0.53
Physical Fitness Self-Assessment IFIS (score 0–25)	0.34	0.03 *
Fear of falling FES-I (score 16–64)	−0.25	0.12

Pearson correlations were used for variables assuming a parametric distribution (IFIS-T, FES-T, weight, height), and Spearman’s rho for non-parametric variables (age, disease duration, PDQ-8). * *p* < 0.05: statistical significance.

## Data Availability

The raw data supporting the conclusions of this article will be made available by the authors on request.
